# Exploration of Synergistic Pesticidal Activities, Control Effects and Toxicology Study of a Monoterpene Essential Oil with Two Natural Alkaloids

**DOI:** 10.3390/toxins15040240

**Published:** 2023-03-25

**Authors:** Jianwei Xu, Min Lv, Shanshan Fang, Yanyan Wang, Houpeng Wen, Shaoyong Zhang, Hui Xu

**Affiliations:** 1 College of Plant Protection, Northwest A&F University, Xianyang 712100, China; 2 Key Laboratory of Vector Biology and Pathogen Control of Zhejiang Province, College of Life Science, Huzhou University, Huzhou 313000, China

**Keywords:** matrine, 1,8-cineole, ternary complex, pesticidal activity, detoxification enzymes, SEM

## Abstract

With the increasing development of pest resistances, it is not easy to achieve satisfactory control effects by using only one agrochemical. Additionally, although the alkaloid matrine (**MT**) isolated from *Sophora flavescens* is now utilized as a botanical pesticide in China, in fact, its pesticidal activities are much lower in magnitude than those of commercially agrochemicals. To improve its pesticidal activities, here, the joint pesticidal effects of **MT** with another alkaloid oxymatrine (**OMT**) (isolated from *S. flavescens*) and the monoterpene essential oil 1,8-cineole (**CN**) (isolated from the eucalyptus leaves) were investigated in the laboratory and greenhouse conditions. Moreover, their toxicological properties were also studied. Against *Plutella xylostella,* when the mass ratio of **MT** and **OMT** was 8/2, good larvicidal activity was obtained; against *Tetranychus urticae,* when the mass ratio of **MT** and **OMT** was 3/7, good acaricidal activity was obtained. Especially when **MT** and **OMT** were combined with **CN**, the significant synergistic effects were observed: against *P. xylostella,* the co-toxicity coefficient (CTC) of **MT**/**OMT** (8/2)/**CN** was 213; against *T. urticae,* the CTC of **MT**/**OMT** (3/7)/**CN** was 252. Moreover, the activity changes over time of two detoxification enzymes, carboxylesterase (CarE) and glutathione *S*-transferase (GST) of *P. xylostella* treated with **MT**/**OMT** (8/2)/**CN**, were observed. In addition, by scanning electron microscope (SEM), the toxicological study suggested that the acaricidal activity of **MT**/**OMT** (3/7)/**CN** may be related to the damage of the cuticle layer crest of *T. urticae*.

## 1. Introduction

*Plutella xylostella* Linnaeus (diamondback moth; Lepidoptera: Plutellidae) as the major migratory insect pest mainly endangered cruciferous vegetables and caused economic loss in the world of up to US$ 4–5 billion annually [1,2]. *P. xylostella* has strong reproductive ability and strong stress resistance. Currently, it has been proved that *P. xylostella* has resistance to a variety of commercial insecticides, which makes it extremely difficult to control [3]. *Tetranychus urticae* Koch (Acari: Tetranychidae) as the major miscellaneous feeding mite had the characteristics of the wide host and high reproduction rate [4,5]. In recent years, with the continuous expansion of vegetable cultivation area, the damage of *T. urticae* becomes more and more serious. The role of chemical insecticides in the management of these pests was indisputable; however, the extensive and irrational use of chemical agrochemicals has resulted in increasing resistances and environmental problems [6,7,8]. Cantharidin and (S)-(-)-palasonin showed different toxicity to *P. xylostella,* with median lethal concentrations, and quinolizidine alkaloids and neem-based products exhibited moderate acaricidal activities against *T. urticae* [9,10,11]. Therefore, the research and development of potential pesticide alternatives from bioactive plant natural products has received much attention in recent years [12,13,14,15,16].

**Figure 1:**
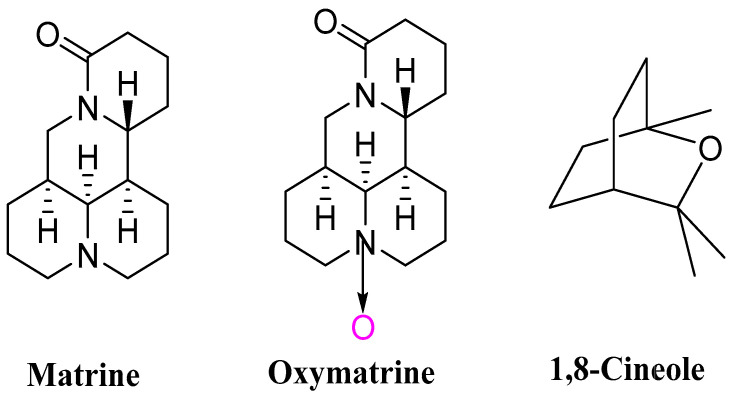
Chemical structures of matrine, oxymatrine and 1,8-cineole.

Matrine (**MT**, Figure 1) and oxymatrine (**OMT**, Figure 1) were two main alkaloids isolated from the plant *Sophora flavescens* (Kushen). Matrine was one of the promising botanical pesticides registered in China due to its good insecticidal, antibacterial, and other agricultural activities [17,18,19,20]. Oxymatrine showed a wide range of biological properties, such as pesticidal [21,22], antiviral [23], anti-inflammatory [24], and anti-tumor effects [25]. The monoterpene 1,8-cineole (**CN**, Figure 1, eucalyptol), an essential oil isolated from the eucalyptus leaves, exhibited a variety of interesting activities, such as antimicrobial [26,27], anti-inflammatory [28], and insecticidal properties [29,30]. The plant essential oils used as botanical pesticides for pest control will be a trend in the foreseeable future [31,32,33,34,35,36,37,38]. Due to their synergistic effects, a mixture of two or three bioactive compounds could play the vital role for the management of pests [39,40,41,42,43,44,45,46]. The unique advantages of plant essential oils can greatly reduce the dosage of pesticides and effectively improve their efficacy level [47,48]. Due to the problems of poor efficiency of matrine and oxymatrine, the wide application of matrine and oxymatrine as the pesticide is restricted. Therefore, in this paper, the pesticidal activities of the different complexes of 1,8-cineole with matrine and oxymatrine were investigated against *P. xylostella* and *T. urticae*. Meanwhile, detoxification enzymes in insects usually play an important role in the process of metabolizing pesticides and producing drug resistance [49]. The change in detoxification activity level may be a response to environmental stress [50]. Carboxylesterase (CarE) and glutathione *S*-transferase (GST) are two important metabolic detoxification enzymes in insects. Moreover, the epidermis, a common component of insect body walls, is an extracellular matrix released by dermal cells and plays a crucial role in maintaining insect body form, reducing water loss and resisting microbial infection and predation [51]. So, the enzyme activity changes over time of CarE and GST of treated *P. xylostella*, and the effects of compounds on the cuticles of *T. cinnabarinus* were also investigated.

## 2. Materials and Methods

### 2.1. Insects and Chemicals

*Tetranychus urticae* (female adults) were reared for many generations in our laboratory with cowpea seedlings as the host plants to establish a stable population (temperature: 26 ± 1 °C; RH: 60–80%; photoperiod: light/dark = 14/10 h). *Plutella xylostella* (3rd instar larvae) were obtained from a laboratory population reared for many generations in the School Key Laboratory of Applied Entomology, Northwest A&F University. The cabbage net seedlings as the host plants were continuously planted in the greenhouse of our laboratory (temperature: 25 ± 2 °C; RH: 70 ± 10%; photoperiod: light/dark = 16/8 h). The host plants did not contact any pesticides. **MT** and **OMT** (98% purity) were purchased from Baoji Haoxiang Biotechnology Co. Ltd. (Baoji, China). **CN** (99% purity) was purchased from Aladdin Chemistry Co., Ltd. (Shanghai, China). β-Cypermethrin (96.6% purity) was bought from Hubei Supur Chemical Co., Ltd. (Hubei, China). Spirodiclofen (98.2% purity) was purchased from Shanghai Yuanye Biotechnology Co., Ltd. (Shanghai, China).

### 2.2. Biological Assay

#### 2.2.1. Insecticidal Activity of Matrine (**MT**), Oxymatrine (**OMT**) and Their Binary Complexes against *P. xylostella* by the Leaf-Loading Poison Method

The solutions of matrine (**MT**), oxymatrine (**OMT**) and their binary complexes were prepared in acetone at 10 mg/mL (toosendanin as the positive control and acetone as CK) (Table 1). For each compound, 45 robust third instar larvae of *P. xylostella* were selected out (15 insects per group). The corresponding solution (1 μL) was evenly spread on a cabbage leaf disc (surface area: 0.25 cm^2^). One piece of the above discs was added and eaten up by each *P. xylostella*, which was raised in each well of 12-well culture plates during 48 h (temperature: 25 ± 2 °C; RH: 70 ± 10%; photoperiod: light/dark = 16/8 h). Their corrected mortality rate (CMR) values (%) = (*T* − *C*) × 100/(100% − *C*); *C* is the mortality rate of CK, and *T* is the mortality rate of the treated *P. xylostella* [52].

#### 2.2.2. Insecticidal Activity of **MT**, **OMT**, **MT**/**OMT** (8/2), 1,8-Cineole (**CN**) and **MT**/**OMT** (8/2)/**CN** against *P. xylostella* by the Leaf-Dipping Method

**Figure 2:**
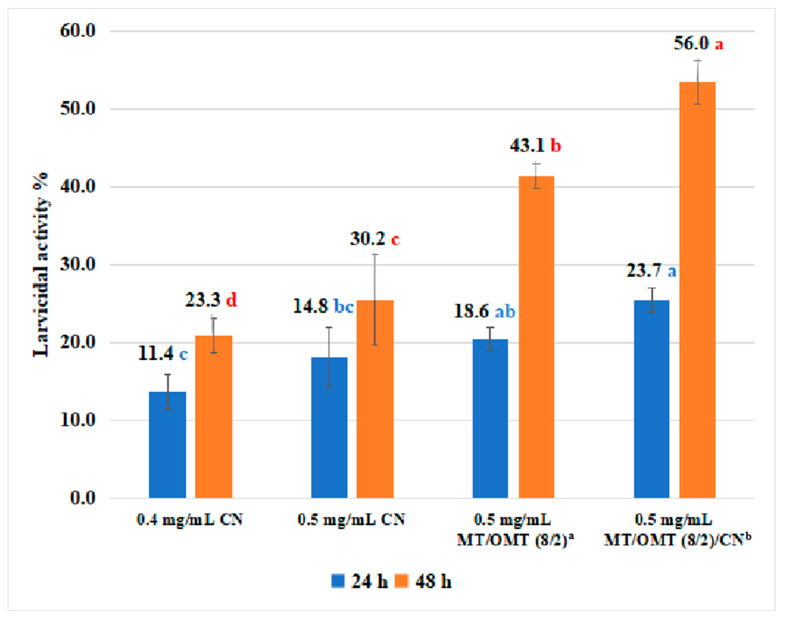
Larvicidal activity of 1,8-cineole (**CN**, 0.4 and 0.5 mg/mL), **MT**/**OMT** (8/2, 0.5 mg/mL) ^*a*^, and **MT**/**OMT** (8/2, 0.5 mg/mL)/**CN** ^*b*^ against *P. xylostella.* ^*a*^ Mass ratio; ^*b*^ **MT**/**OMT** (mass ratio: 8/2) were dissolved in the solution of **CN** (C**_CN_**= 0.4 mg/mL) in acetone. Multiple range test using Duncan’s test (*p* < 0.05). The same letters denote treatments that are not significantly different from each other (different compounds at the same time).

The procedure for evaluation of larvicidal activity of 1,8-cineole (**CN**), **MT**/**OMT** (8/2), and **MT**/**OMT** (8/2)/**CN** against *P. xylostella* (Figure 2) was as follows: The solutions of 1,8-cineole (**CN**, 0.4 and 0.5 mg/mL), **MT**/**OMT** (8/2, 0.5 mg/mL), and **MT**/**OMT** (8/2, 0.5 mg/mL)/**CN** (C**_CN_**= 0.4 mg/mL) were prepared in acetone (acetone was used as CK). For each compound, 45 robust third instar larvae of *P. xylostella* were selected (15 insects per group). The cabbage leaf disc (surface area: 0.25 cm^2^) was dipped into the corresponding solution for 3 s and taken out. The treated ones were added to three dishes during 48 h (15 insects per dish) (temperature: 25 ± 2 °C; RH: 70 ± 10%; photoperiod: light/dark = 16/8 h). Their CMR values were calculated in the same way as mentioned above [53].

The procedure for the determination of LC_50_ values of **MT**, **OMT**, **MT**/**OMT** (8/2), and **MT**/**OMT** (8/2)/**CN** at 48 h against *P. xylostella* was as follows (Table 2): Firstly, five different concentrations (2, 1, 0.5, 0.25, and 0.125 mg/mL) of **MT**, **OMT**, **MT**/**OMT** (8/2), and **MT**/**OMT** (8/2)/**CN** were prepared in acetone (acetone was used as CK). Five concentrations of β-cypermethrin (a positive control) were set as 0.5, 0.25, 0.125, 0.0625, and 0.03125 mg/mL in acetone, respectively. For each concentration, 45 robust third instar larvae of *P. xylostella* were selected (15 insects per group). The cabbage leaf disc (surface area: 0.25 cm^2^) was dipped into the corresponding solution for 3 s and taken out. The treated ones were added to three dishes during 48 h (15 insects per dish) (temperature: 25 ± 2 °C; RH: 70 ± 10%; photoperiod: light/dark = 16/8 h). Their CMR values at 48 h were calculated in the same way as mentioned above. Finally, their 48 h median lethal concentration (LC_50_) values were calculated upon the different concentrations and CMRs (Table 3).

The co-toxicity coefficient (CTC) values of the binary complexes were further evaluated according to Sun’s formula [54]. The value of CTC is used to determine whether the efficiency is increased: when CTC > 120, it is synergistic; when CTC < 80, it is antagonistic; when 80 < CTC < 120, it is additive. A significant synergistic effect is observed when the value of CTC is 200.

#### 2.2.3. Control Efficiency of **MT**/**OMT** (8/2), and **MT**/**OMT** (8/2)/**CN** against *P. xylostella* in the Greenhouse

The solutions of **MT**/**OMT** (8/2), **MT**/**OMT** (8/2)/**CN** and β-cypermethrin were prepared at 0.2 mg/mL in 0.1% aq. Tween-80, respectively (Table 4). Each cabbage seedling was infested with 20 third instar larvae of *P. xylostella* prior to spraying. One cabbage seedling was chosen for one group, and each treatment was three replicates. An airbrush was used to spray 10 mL of the corresponding solution for each treatment. The cabbage seedlings treated with 0.1% aq. Tween-80 alone were used as CK (temperature: 25 ± 2 °C; RH: 70 ± 10%; photoperiod: light/dark = 16/8 h). Their control effects on the 1st, 3rd, and 5th days were calculated in the same way as mentioned above [55].

#### 2.2.4. Acaricidal Activity of **MT**, **OMT**, **CN**, and Their Mixtures against *T. urticae* by the Slide-Dipping Method

The solutions of **MT**, **OMT**, their binary complexes (with different mass ratio), and spirodiclofen (a positive control) (treated by 0.1 g/L of aq. Tween-80 as CK) were prepared at 0.5 mg/mL in Tween-80 in water (0.1 g/L), respectively (Table 5). For each compound, 90–120 healthy and size-consistency female adults of mites (30–40 ones per group) were selected out. Slides affixed with mites were dipped into the corresponding solution for 5 s and taken out (temperature: 26 ± 1 °C; RH: 60–80%; photoperiod: light/dark = 14/10 h). Their mortalities at 48 and 72 h were calculated as follows: corrected mortality rate (%) = (*T* − *C*) × 100/(100% − *C*); *C* is the mortality rate of CK, and *T* is the mortality rate of the treated *T. urticae* [56].

According to the above results, the acaricidal activity of **CN** and **MT**/**OMT** (3/7)/**CN** against *T. urticae* (Table 5) was tested as follows: The solution of **CN** was prepared at 0.048 mg/mL in Tween-80 in water (0.1 g/L), and the solution of **MT**/**OMT** (3/7)/**CN** was prepared at 0.5 mg/mL in Tween-80 in water (0.1 g/L) containing **CN** (C**_CN_**= 0.048 mg/mL). The next procedure was completed in the same way as mentioned above.

#### 2.2.5. LC_50_ of **MT**, **OMT**, **MT**/**OMT** (3/7), and **MT**/**OMT** (3/7)/**CN** against *T. urticae*

Firstly, five different concentrations (6, 3, 1.5, 0.75, and 0.375 mg/mL) of **MT** and **OMT** were prepared in Tween-80 in water (0.1 g/L) (treated by 0.1 g/L of aq. Tween-80 as CK) (Table 6). Six concentrations of **MT**/**OMT** (3/7) and **MT**/**OMT** (3/7)/**CN** were set as 4, 2, 1, 0.5, 0.25, and 0.125 mg/mL in Tween-80 in water (0.1 g/L), respectively. Five concentrations of spirodiclofen were set as 0.5, 0.25, 0.125, 0.0625, and 0.03125 mg/mL in Tween-80 in water (0.1 g/L), respectively. For each concentration, 90–120 healthy and size-consistency female adults of mites (30–40 ones per group) were selected out. Slides affixed with mites were dipped into the corresponding solution for 5 s and taken out (temperature: 26 ± 1 °C; RH: 60–80%; photoperiod: light/dark = 14/10 h). Their CMR values at 72 h were calculated in the same way as mentioned above. Finally, LC_50_ values were calculated by the linear regressions of 72 h CMRs (%) and concentrations (Table 7) [56]. The co-toxicity coefficient (CTC) values of the binary complexes were further evaluated according to Sun’s formula [54].

#### 2.2.6. Control Efficiency of **MT**/**OMT** (3/7), and **MT**/**OMT** (3/7)/**CN** against *T. urticae* in the Greenhouse

The solutions of **MT**/**OMT** (3/7), **MT**/**OMT** (3/7)/**CN**, and spirodiclofen were all prepared at 0.2 mg/mL in 0.1% aq. Tween-80, respectively. Asparagus bean plants were infested with the female adults of *T. urticae* prior to spraying. Three plants were chosen for one group, and each treatment was three replicates. An airbrush was used to spray 10 mL of the corresponding solution for three replicates. The plants treated with 0.1% aq. Tween-80 alone were used as CK (temperature: 26 ± 1 °C; RH: 60–80%; photoperiod: light/dark = 14/10 h). Their control effects on the 1st, 3rd, and 5th days were calculated in the same way as mentioned above (Table 8) [56].

### 2.3. Enzyme Activity Assay against P. xylostella

#### 2.3.1. Sample Preparation Using Leaf-Dipping Method

According to the above-mentioned leaf-dipping method, 180 robust 3rd instar larvae of *P. xylostella* were treated with **CN**, **MT**/**OMT** (8/2) and **MT**/**OMT** (8/2)/**CN** at 0.4 mg/mL, respectively (treated by acetone as CK). Then, the 30 surviving larvae in the treated group were collected at 12, 24, 36, and 48 h, respectively. They were then snap-frozen in liquid nitrogen and stored at −80 °C for subsequent enzyme activity analysis [53].

#### 2.3.2. Preparation of Homogenous Liquid

The homogenization treatment (including ten larvae: Weight (g)/Volume (mL) = 1/10) was performed in an ice bath. The homogenous liquid was obtained for carboxylesterase (CarE) activity assay when the sample was centrifuged at 12,000× *g* for 30 min at 4 °C. The homogenous liquid was obtained for glutathione-*S*-transferase (GST) activity assay when the sample was centrifuged at 8000× *g* for 10 min at 4 °C.

#### 2.3.3. CarE and GST Activity Assay According to Bradford’s Method

The absorption values over time of CarE and GST were tested by using CarE (α-naphthyl acetate (α-NA) as a substrate) and GST (1-chloro-2,4-dinitrobenzene and reduced glutathione as substrates) assay kits (Suzhou Keming Biotechnology Co., Ltd., China), respectively. Total protein concentration was determined according to the Bradford method (using bovine serum albumin (BSA) as a standard). The protein content was tested by a BCA protein quantitative assay kit (Shaanxi Zhonghui Hecai Biomedical Technology Co., Ltd., China). Finally, the enzymes activity values were obtained according to the absorption value and the protein content. Each treatment was replicated three times [57].

### 2.4. Analysis of Morphology of Cuticles

#### 2.4.1. Pretreatment of Mites

The complex (**MT**/**OMT** (3/7)/**CN**) was prepared at 0.894 mg/mL in 0.1 g/L of aq. Tween-80 (0.1 g/L of aq. Tween-80 as CK). Female adult mites with the same physiological status and good growth conditions were selected to cowpea leaves (5 cm in length and 3 cm in width; 30 ones/leaf). After 4 h, the leaves were immersed in the above solution for 5 s, the excess solution was absorbed by filter paper, and the leaves were placed in a Petri dish with a moist sponge (three replicates/treatment). Then, Petri dishes were placed in a light incubator (temperature: 26 ± 1 °C; RH: 60–80%; photoperiod: light/dark = 14/10 h). Finally, the dead mites were collected at 24, 48 and 72 h after treatment by the complex for scanning electron microscope (SEM) analysis [56,58].

#### 2.4.2. Observation of Morphology and Structural Changes of Cuticles in Spider Mites

The collected samples were fixed with 2.5% glutaraldehyde under ice bath conditions, incubated at 4 °C for 4 h, rinsed three times with 0.1 mol/L of phosphate buffer saline (PBS), dehydrated with different concentrations of ethanol, and freeze-dried for 3 h. The mites were then placed on a sample table and sprayed with gold under vacuum conditions. The morphology was observed and photographed by S-3400N SEM [59].

### 2.5. Statistic Analysis

Mortality data were corrected with Abbott’s formula and analyzed by a multiple range test using Duncan’s test (*p* < 0.05). The median lethal concentration (LC_50_) values were calculated on log-concentration versus probit (% mortality) regression analysis. The values of r, χ^2^, df, and *P* were obtained on regression analysis by IBM SPSS Statistics 20.0 (*p* < 0.05).

## 3. Results and Discussion

### 3.1. Insecticidal Activity

**Figure 3:**
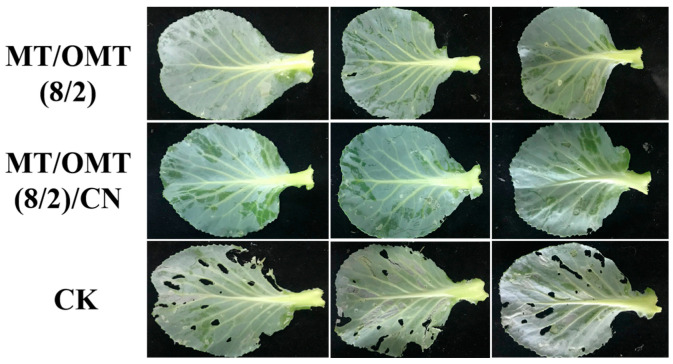
Fifth-day symptoms of cabbage seedling leaves treated with **MT**/**OMT** (8/2, 0.2 mg/mL) and **MT**/**OMT** (8/2, 0.2 mg/mL)/**CN** against *P. xylostella*.

First, the insecticidal activities of two compounds (**MT** and **OMT**) and their binary mixtures at different mass ratios against *P. xylostella* were tested. As shown in Table 1, to binary mixtures, when the mass ratio of **MT** and **OMT** was 8:2, the corresponding 48 h corrected mortality rate (CMR) was 27.9%, which was higher than those of **MT** (20.9%) and **OMT** (16.2%). The 48 h CMRs were all 20.9% when the mass ratio of **MT**/**OMT** was 7:3 or 5:5. The 48 h CMR was 16.2% when the mass ratio of **MT**/**OMT** was 1:9, which was similar to that of **OMT**. However, when the mass ratio of **MT** and **OMT** was 3:7, the corresponding 48 h CMR was decreased to 13.9%, which was lower than those of **MT** and **OMT**. Obviously, for **MT** and **OMT** against *P. xylostella*, the best mass ratio of **MT** and **OMT** was 8:2. As shown in Figure 2, against *P. xylostella*, 48 h CMRs of 1,8-cineole (**CN**, 0.4 and 0.5 mg/mL), **MT**/**OMT** (8/2, 0.5 mg/mL), and **MT**/**OMT** (8/2, 0.5 mg/mL)/**CN** were 23.3%, 30.2%, 43.1%, and 56.0%, respectively. It demonstrated that when **CN** (0.4 mg/mL) was combined with **MT**/**OMT**(8/2, 0.5 mg/mL), the larvicidal activity of the corresponding complex was significantly improved. Then, 48 h LC_50_ values of **MT**, **OMT**, **MT**/**OMT** (8/2), and **MT**/**OMT** (8/2)/**CN** against *P. xylostella* were further evaluated according to their CMRs at different concentrations (Table 2). As described in Table 3, 48 h LC_50_ values of **MT**/**OMT** (8/2), and **MT**/**OMT** (8/2)/**CN** were 0.708 and 0.441 mg/mL, respectively; while 48 h LC_50_ values of **MT** and **OMT** were 0.899 and 1.158 mg/mL, respectively. That is, the larvicidal activity of **MT**/**OMT** (8/2)/**CN** was 2.0–2.6 fold those of **MT** and **OMT**. Moreover, the co-toxicity coefficient (CTC) values of **MT**/**OMT** (8/2) and **MT**/**OMT** (8/2)/**CN** were 133 and 213, respectively. Clearly, their CTCs were all > 120, so the above two mixtures showed the synergistic effect. Subsequently, the control effects of **MT**/**OMT** (8/2) and **MT**/**OMT** (8/2)/**CN** against *P. xylostella* in the greenhouse at 0.2 mg/mL were tested. As shown in Table 4, the control effects of **MT**/**OMT** (8/2) and **MT**/**OMT** (8/2)/**CN** against *P. xylostella* after 5 days were 36.8% and 49.0%, respectively. Symptoms at the fifth day of the cabbage seedling leaves treated with **MT**/**OMT** (8/2) and **MT**/**OMT** (8/2)/**CN** against *P. xylostella* are described in Figure 3. In the **MT**/**OMT** (8/2)- and **MT**/**OMT** (8/2)/**CN**-treated groups, the surfaces of the leaves were smooth and had almost no small puncture; however, there were many small punctures eaten by *P. xylostella* on the leaves in the CK-treated group. Photographs of control effects of those complexes against *P xylostella* are shown in Figures S1–S3.

### 3.2. Acaricidal Activity

**Figure 4:**
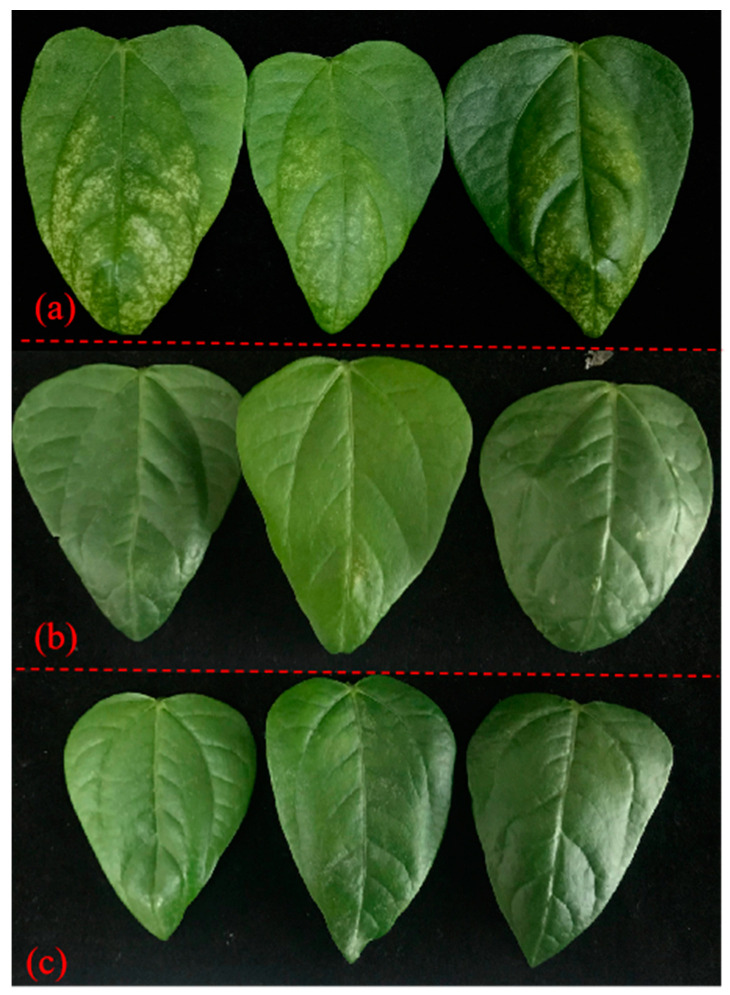
Fifth-day symptoms of asparagus bean seedling leaves treated with **CK** (**a**), **MT**/**OMT** (3/7, 0.2 mg/mL) (**b**), and **MT**/**OMT** (3/7, 0.2 mg/mL)/**CN** (**c**) against *T. urticae*.

The 48 and 72 h acaricidal results of **MT**, **OMT**, and their binary mixtures at 0.5 mg/mL against the female adults of *T. urticae* are shown in Table 5. Among nine binary complexes, the 72 h CMRs of six complexes were higher than those of **MT** (23.5%) and **OMT** (18.2%); especially when the mass ratio of **MT** to **OMT** was 3:7, the corresponding 72 h CMR was 34.8%. At 0.048 mg/mL, the 72 h CMR of **CN** against *T*. *urticae* was only 11.2%; interestingly, when **MT** and **OMT** (mass ratio: 3/7) were dissolved in the solution of **CN** (C**_CN_**= 0.048 mg/mL) in 0.01% aq. Tween-80, and C**_MT_**_/**OMT** (3/7)_ was 0.5 mg/mL, the corresponding CMR was increased to 38.0%. It may be related to the strong permeability of **CN** as an essential oil [38]. Then, 72 h LC_50_ values of **MT**, **OMT**, **MT**/**OMT** (3/7), and **MT**/**OMT** (3/7)/**CN** against *T*. *urticae* were further calculated according to their CMRs at different concentrations (Table 6). As shown in Table 7, the 72 h LC_50_ values of **MT**/**OMT** (3/7) and **MT**/**OMT** (3/7)/**CN** (C**_CN_**= 0.048 mg/mL) were 1.405 and 0.894 mg/mL, respectively, while 72 h LC_50_ values of **MT** and **OMT** were 2.014 and 2.377 mg/mL, respectively. That is, the acaricidal activity of **MT**/**OMT** (3/7)/**CN** (C**_CN_**= 0.048 mg/mL) was increased to 2.3–2.7 folds when compared with those of **MT** and **OMT**. Meanwhile, the CTCs of **MT**/**OMT** (3/7) and **MT**/**OMT** (3/7)/**CN** (C**_CN_**= 0.048 mg/mL) were 160 and 252, respectively. So, the above two mixtures showed the synergistic effect especially for the combination of **MT**/**OMT** (3/7)/**CN**. On the other hand, the control efficiency of **MT**/**OMT** (3/7) and **MT**/**OMT** (3/7)/**CN** (C**_CN_** = 0.04 mg/mL in 0.1% aq. Tween-80) at 0.2 mg/mL against *T*. *urticae* in the greenhouse was evaluated. As shown in Table 8, the control effects of **MT**/**OMT** (3/7) and **MT**/**OMT** (3/7)/**CN** after 5 days were 31.3% and 42.3%, respectively. Fifth-day symptoms of asparagus bean seedling leaves treated with **MT**/**OMT** (3/7) and **MT**/**OMT** (3/7)/**CN** were described in Figure 4. There were lots of white spots destroyed by *T*. *urticae* on the seedling leaves in the CK-treated group, whereas in the **MT**/**OMT** (3/7)- and **MT**/**OMT** (3/7)/**CN**-treated groups, almost no small white spots were on the seedling leaves. These findings were the same as our previous report [58]. Photographs of control effects of those complexes against *T*. *urticae* are shown in Figures S4–S6.

### 3.3. Changes of Detoxification Enzymes Activities

**Figure 5:**
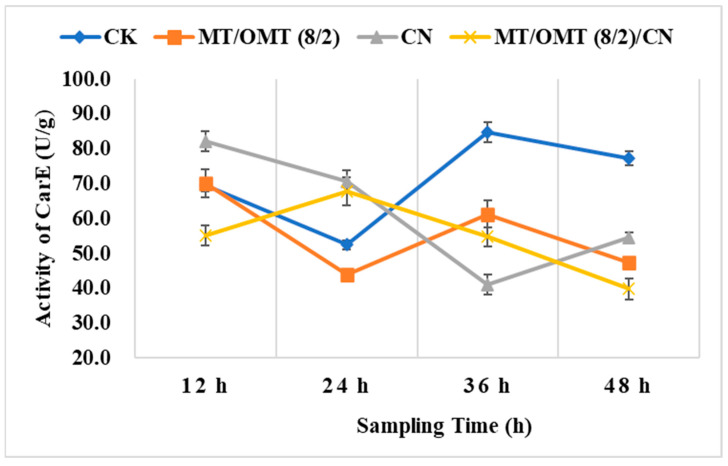
Effects of **CN**, **MT**/**OMT** (8/2) and **MT**/**OMT** (8/2)/**CN** at 0.4 mg/mL on CarE in *P. xylostella* at different sampling times..

**Figure 6:**
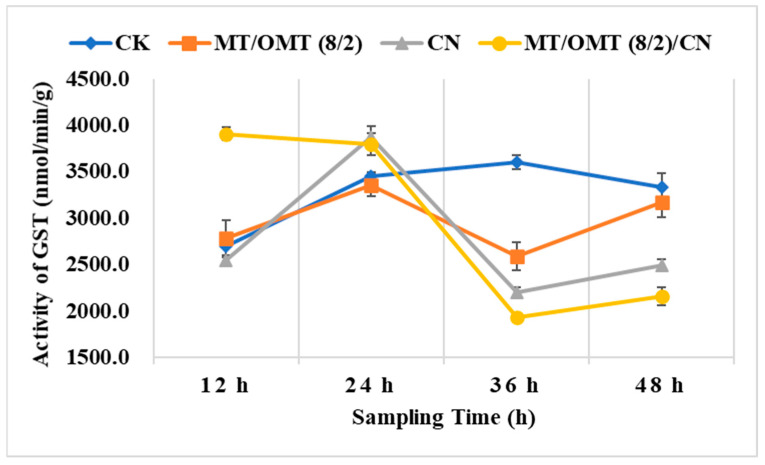
Effects of **CN**, **MT**/**OMT** (8/2) and **MT**/**OMT** (8/2)/**CN** at 0.4 mg/mL on GST in *P. xylostella* at different sampling times.

Subsequently, the changes of detoxification enzymes (CarE and GST) activities in *P. xylostella,* treated with **CN**, **MT**/**OMT** (8/2) and **MT**/**OMT** (8/2)/**CN** at 0.4 mg/mL after 12, 24, 36, and 48 h were depicted in Figure 5 and Figure 6, respectively. The enzymatic activities of CarE and GST are always in dynamic change. As described in Figure 5, the CarE activity values in the treated groups at 36 and 48 h were much lower than those of the control group. The CarE activity value in the **CN**-treated group at 12 h (82.1 U/g) was higher than that of the control group (69.4 U/g). It suggested that **CN** was toxic to *P. xylostella* and stimulated the detoxification ability of the CarE in *P. xylostella* at 12 h. At 24 h, the CarE activity value in the **MT**/**OMT** (8/2)/**CN**-treated group (67.7 U/g) was higher than that of the control group (52.4 U/g). It indicated that **MT**/**OMT** (8/2)/**CN** stimulated the detoxification ability of the CarE in *P. xylostella* at 24 h. After that, the detoxification ability of the CarE in *P. xylostella* in all treated groups was inhibited. The time for reaching the lowest points of the CarE activities in the treated groups was different. For example, in the **CN**-treated group, the lowest point of the CarE activity was reached at 36 h, whereas the lowest points of the CarE activity in the **MT**/**OMT** (8/2)- and **MT**/**OMT** (8/2)/**CN**-treated group were reached at 48 h.

As illustrated in Figure 6, the GST activity value in the **MT**/**OMT** (8/2)/**CN**-treated group at 12 h (3910 nmol/min/g) was higher than that of the control group (2698 nmol/min/g). Similarly, it suggested that the mixture **MT**/**OMT** (8/2)/**CN** stimulated and enhanced the detoxification ability of the GST in *P. xylostella* at 12 h. At 24 h, the GST activity value in the **CN**-treated group was slightly higher than that of the control group. Afterwards, the GST activity in the treated groups decreased significantly when compared with that of the control, and it reached the lowest point at 36 h in all treated groups. The GST activity values at 36 h in **CN-**, **MT**/**OMT** (8/2)- and **MT**/**OMT** (8/2)/**CN**-treated groups were 2208, 2591 and 1932 nmol/min/g, respectively, and they were decreased 1.4–1.9 folds of that of the control group (3603 nmol/min/g). Obviously, the GST activity of *P. xylostella* in the treated groups was largely inhibited at 36 h.

### 3.4. Toxicological Study of Structural Changes of Cuticles by SEM

**Figure 7:**
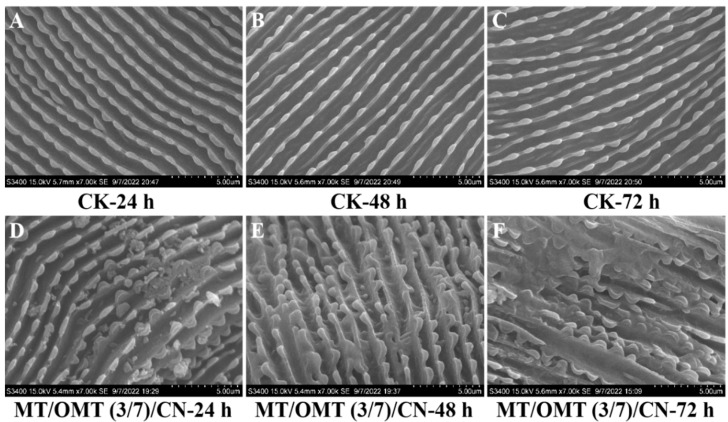
Scanning electron microscope images of cuticles of *T. urticae* treated with **MT/OMT** (3/7)**/CN** (**D**–**F**) and aq. Tween-80 (0.1 g/L) (CK) (**A**–**C**) after 24, 48 and 72 h, respectively. Bars: 5.0 µm.

The penetration of insecticide to pest epidermis is the premise of its toxic effect. The cuticle serves as the initial barrier of defense between the body and the outside world, effectively blocking pesticide penetration. The reduction in epidermal penetration can delay the time of pesticide reaching the target site, and pests have sufficient time and faster speed to metabolize the pesticide entering the body [59]. As shown in Figure 7, in the CK-treated group, the cuticles of *T. urticae* are flat and have an entire structure, with neat and continuous skin texture; whereas in the **MT/OMT** (3/7)**/CN**-treated group, the structure of the cuticles was damaged with visible wrinkles and an uneven arrangement of the inner ridges. The cuticles of wounded mites were thinner, softer, and more asymmetrically bent than those of normal mites, losing their barrier-like function against acaricide penetration. As a result, **CN** may enhance the penetration ability of this complex on *T. urticae* [38,60].

## 4. Conclusions

In summary, the significant synergistic effects were observed when **CN** was combined with **MT** and **OMT** as pesticidal agents: against *P. xylostella,* the CTC of **MT**/**OMT** (8/2)/**CN** was 213; against *T. urticae,* the CTC of **MT**/**OMT** (3/7)/**CN** was 252. Furthermore, these mixtures displayed good control efficiency against *P. xylostella* and *T. urticae* in the greenhouse. Importantly, the enzymes activity changes over time of CarE and GST in *P. xylostella* treated with **CN**, **MT**/**OMT** (8/2) and **MT**/**OMT** (8/2)/**CN** were explored. Due to the accumulation of toxicant leading to a decrease in the detoxification ability, the time for reaching the lowest point of the CarE activity in the treated groups was different, whereas the time for reaching the lowest point of the GST activity in the treated groups was the same (at 36 h). Notably, by SEM analysis, the toxicology study suggested that the destruction of the cuticle layer crest of *T. urticae* by **MT**/**OMT** (3/7)/**CN** may be the main cause of their death. These results will pave the way for the future study of different combinations of **MT**–**OMT**–**CN** and the application of **CN** as a synergist with other bioactive natural products as pesticidal candidates in crop protection.

## Figures and Tables

**Table 1:** Insecticidal activity of matrine (**MT**), oxymatrine (**OMT**) and their binary mixtures against *P. xylostella* at 10 μg/larva.

Compound	Corrected Mortality Rate (Mean ± SE, %)	
	24 h	48 h
**MT**	9.1 ± 2.2	20.9 ± 2.2 b *^b^*
**MT/OMT** (9/1) ^*a*^	11.3 ± 0	16.2 ± 0 bc
**MT/OMT** (8/2)	13.6 ± 2.2	27.9 ± 2.2 a
**MT/OMT** (7/3)	11.3 ± 3.8	20.9 ± 2.2 b
**MT/OMT** (6/4)	2.3 ± 2.2	18.6 ± 2.2 bc
**MT/OMT** (5/5)	4.5 ± 0	20.9 ± 2.2 b
**MT/OMT** (4/6)	9.1 ± 2.2	18.6 ± 2.2 bc
**MT/OMT** (3/7)	4.5 ± 0	13.9 ± 2.2 c
**MT/OMT** (2/8)	2.3 ± 2.2	18.6 ± 2.2 bc
**MT/OMT** (1/9)	4.5 ± 0	16.2 ± 0 bc
**OMT**	2.3 ± 2.2	16.2 ± 0 bc
toosendanin	11.3 ± 0	32.5 ± 2.2 a

*^a^* Mass ratio; ^*b*^ Multiple range test using Duncan’s test (*p* < 0.05). The same letters denote treatments that are not significantly different from each other.

**Table 2:** Corrected mortality rates at 48 h at different concentrations of **MT**, **OMT**, **MT**/**OMT** (8/2), and **MT**/**OMT** (8/2)/1,8-cineole (**CN**) against *P*. *xylostella*.

Compound	Corrected Mortality Rate (%) at Concentration (mg/mL) *^a^*				
	2 (0.5) *^b^*	1 (0.25) *^b^*	0.5 (0.125) *^b^*	0.25 (0.0625) *^b^*	0.125 (0.03125) *^b^*
**MT**	65.1 ± 3.8	53.4 ± 2.2	39.5 ± 2.2	23.2 ± 0	13.9 ± 2.2
**OMT**	60.4 ± 4.4	48.8 ± 2.2	32.5 ± 4.4	18.6 ± 2.2	11.6 ± 2.2
**MT/OMT** (8/2)^*c*^	72.4 ± 3.3	58.6 ± 0	41.3 ± 1.6	27.5 ± 2.8	15.5 ± 1.6
**MT/OMT** (8/2)**/CN**^*d*^	81.0 ± 1.6	65.5 ± 1.6	53.4 ± 2.8	37.9 ± 0	24.1 ± 1.6
β-cypermethrin	83.7 ± 2.2	55.8 ± 5.8	37.2 ± 3.8	25.5 ± 2.2	13.9 ± 2.2

*^a^* Values are mean ± SE of three replicates. *^b^* Number in parentheses is the tested concentration of β-cypermethrin. *^c^* Mass ratio; *^d^* **MT**/**OMT** (mass ratio: 8/2) containing **CN** (C**_CN_**= 0.4 mg/mL) in acetone.

**Table 3:** LC_50_ values of **MT**, **OMT**, **MT**/**OMT** (8/2), and **MT**/**OMT** (8/2)/**CN** at 48 h against *P. xylostella*.

Compound	Linear Regression Equation^*a*^	LC_50_ (mg/mL)	Confidence Interval 95% (mg/mL)	r	χ^2^	df	*P*	Co-Toxicity Coefficient
**MT**	Y = 0.057 + 1.240X	0.899	0.636~1.455	0.997	0.171	3	0.982	/
**OMT**	Y = −0.08 + 1.258X	1.158	0.806~2.045	0.993	0.155	3	0.984	/
**MT/OMT** (8/2) ^*b*^	Y = −0.201 + 1.338X	0.708	0.539~0.970	0.998	0.025	3	0.999	133
**MT/OMT** (8/2)/ **CN** ^*c*^	Y = 0.456 + 1.280X	0.441	0.324~0.585	0.999	0.161	3	0.984	213
β-cypermethrin	Y = 1.281 + 1.652X	0.168	0.128~0.226	0.980	1.845	3	0.605	/

*^a^* Regression analysis by IBM SPSS Statistics 20.0 (*p* < 0.05); *^b^* Mass ratio; *^c^* **MT**/**OMT** (mass ratio: 8/2) containing **CN** (C**_CN_** = 0.4 mg/mL) in acetone.

**Table 4:** Control efficiency of **MT**/**OMT** (8/2), and **MT**/**OMT** (8/2)/**CN** against *P. xylostella* in the greenhouse tests at a concentration of 0.2 mg/mL.

Compound	Control Efficiency (%) *^c^*		
	1st Day	3rd Day	5th Day
**MT/OMT** (8/2) ^*a*^	10.1 ± 1.6 b *^d^*	25.8 ± 1.6 b	36.8 ± 0 c
**MT/OMT** (8/2)/**CN** ^*b*^	15.5 ± 4.4 b	32.1 ± 3.3 b	49.0 ± 3.3 b
β-cypermethrin	40.6 ± 4.4 a	58.6 ± 2.8 a	71.9 ± 1.6 a

*^a^* Mass ratio; *^b^* **MT** and **OMT** (mass ratio: 8/2) containing **CN** (C**_CN_** = 0.04 mg/mL) in 0.1% aq. Tween-80; *^c^* Values are mean ± SE of three replicates; *^d^* Multiple range test using Duncan’s test (*p* < 0.05). The same letters denote treatments that are not significantly different from each other (different compounds at the same time).

**Table 5:** Acaricidal activity of **MT**, **OMT**, **CN**, and their mixtures against *T. urticae* treated at a concentration of 0.5 mg/mL.

Compound	Corrected Mortality Rate (Mean ± SE, %)	
	48 h	72 h
**MT**	7.0 ± 2.5	23.5 ± 1.7 cde *^d^*
**MT/OMT** (9/1) ^*a*^	10.5 ± 1.6	20.4 ± 3.2 cdef
**MT/OMT** (8/2)	6.9 ± 0.7	24.3 ± 2.3 cde
**MT/OMT** (7/3)	4.5 ± 1.0	21.9 ± 5.8 cdef
**MT/OMT** (6/4)	10.3 ± 0.5	16.0 ± 3.4 ef
**MT/OMT** (5/5)	10.9 ± 1.6	24.6 ± 4.4 cde
**MT/OMT** (4/6)	8.7 ± 2.0	27.1 ± 3.4 bcde
**MT/OMT** (3/7)	9.4 ± 3.2	32.2 ± 0.9 bc
**MT/OMT** (2/8)	9.8 ± 2.2	28.9 ± 1.6 bcd
**MT/OMT** (1/9)	4.0 ± 1.2	25.2 ± 6.3 cde
**OMT**	6.0 ± 1.3	18.2 ± 2.9 def
1,8-cineole (CN) *^b^*	6.5 ± 0.2	11.2 ± 0.4 f
**MT/OMT** (3/7)/**CN** ^*c*^	17.8 ± 0.3	38.0 ± 3.2 b
spirodiclofen	47.7 ± 4.7	88.6 ± 2.3 a

*^a^* Mass ratio; *^b^* at a concentration of 0.048 mg/mL; *^c^* **MT**/**OMT** (mass ratio: 3/7) containing **CN** (C**_CN_**= 0.048 mg/mL) in Tween-80 in water (0.1 g/L); *^d^* Multiple range test using Duncan’s test (*p* < 0.05). The same letters denote treatments that are not significantly different from each other (different compounds at the same time).

**Table 6:** Corrected mortality rates at 72 h at different concentrations of **MT**, **OMT**, **MT**/**OMT** (3/7), and **MT**/**OMT** (3/7)/**CN** against *T. urticae*.

Compound	Corrected Mortality Rate (%) at Concentration (mg/mL) *^a^*					
	6	3	1.5	0.75	0.375	/
**MT**	80.3 ± 0.9	56.6 ± 1.6	40.5 ± 2.4	24.1 ± 2.7	15.6 ± 3.9	/
**OMT**	75.1 ± 2.4	53.9 ± 2.2	38.5 ± 2.1	20.5 ± 3.5	10.3 ± 1.0	/
Compound	Corrected mortality rate (%) at concentration (mg/mL) *^a^*					
	4 (0.5) *^b^*	2 (0.25) *^b^*	1 (0.125) *^b^*	0.5 (0.0625) *^b^*	0.25 (0.03125) *^b^*	0.125
**MT/OMT** (3/7) *^c^*	72.6 ± 0.4	53.2 ± 1.3	40.4 ± 2.7	32.2 ± 0.9	21.7 ± 0.6	13.4 ± 1.5
**MT/OMT** (3/7)/**CN** *^d^*	80.1 ± 0.8	61.0 ± 0.5	49.6 ± 2.2	38.0 ± 3.2	29.6 ± 3.5	17.4 ± 1.6
spirodiclofen	90.1 ± 0.5	72.5 ± 0.2	54.5 ± 0.8	36.8 ± 2.6	26.0 ± 1.2	/

*^a^* Values are mean ± SE of three replicates. *^b^* Number in parentheses is the tested concentration of spirodiclofen. *^c^* Mass ratio; *^d^* **MT** and **OMT** (mass ratio: 3/7) containing **CN** (C**_CN_** = 0.048 mg/mL) in Tween-80 in water (0.1 g/L).

**Table 7:** LC_50_ values of **MT**, **OMT**, **MT**/**OMT** (3/7), and **MT**/**OMT** (3/7)/**CN** at 72 h against *T*. *urticae*.

Compound	Linear Regression Equation ^*a*^	LC_50_ (mg/mL)	Confidence Interval 95% (mg/mL)	r	χ^2^	df	*P*	Co-Toxicity Coefficient
**MT**	Y = −0.471 + 1.549X	2.014	1.667~2.451	0.987	2.004	3	0.572	/
**OMT**	Y = −0.604 + 1.606X	2.377	1.970~2.924	0.994	0.398	3	0.941	/
**MT/OMT** (3/7) ^*b*^	Y = −0.160 + 1.083X	1.405	1.108~1.858	0.986	1.681	4	0.794	160
**MT/OMT** (3/7)/**CN** ^*c*^	Y = 0.054 + 1.103X	0.894	0.713~1.134	0.992	2.166	4	0.705	252
spirodiclofen	Y = 1.597 + 1.558X	0.094	0.077~0.113	0.996	2.198	3	0.532	/

*^a^* Regression analysis by IBM SPSS Statistics 20.0 (*p* < 0.05); *^b^* Mass ratio; *^c^* **MT** and **OMT** (mass ratio: 3/7) containing **CN** (C**_CN_** = 0.048 mg/mL) in Tween-80 in water (0.1 g/L).

**Table 8:** Control efficiency of **MT**/**OMT** (3/7), and **MT**/**OMT** (3/7)/**CN** against *T. urticae* in the greenhouse tests at a concentration of 0.2 mg/mL.

Compound	Control Efficiency (%) *^c^*		
	1st Day	3rd Day	5th Day
**MT/OMT** (3/7) *^a^*	7.7 ± 0.8 b *^d^*	19.6 ± 1.9 c	31.3 ± 1.1 c
**MT/OMT** (3/7)/**CN** *^b^*	13.8 ± 1.8 b	31.9 ± 1.0 b	42.3 ± 1.2 b
spirodiclofen	25.0 ± 2.4 a	58.3 ± 1.0 a	73.9 ± 1.5 a

*^a^* Mass ratio; *^b^* **MT** and **OMT** (mass ratio: 3/7) containing **CN** (C**_CN_** = 0.04 mg/mL) in 0.1% aq. Tween-80; *^c^* Values are mean ± SE of three replicates; *^d^* Multiple range test using Duncan’s test (*p* < 0.05). The same letters denote treatments that are not significantly different from each other (different compounds at the same time).

## Data Availability

The dataset utilized in this study is available upon request.

## Supplementary Materials

The following supporting information can be downloaded at: https://www.mdpi.com/article/10.3390/toxins15040240/s1, Biological assay; Pictures of control effects of different complexes against *P. xylostella*; Pictures of control effects of different complexes against *T. urticae*. Figure S1. Pictures of control efficiency of **CK** after 1st day (a), 3rd day (b) and 5th day (c) against *P. xylostella* in the greenhouse; Figure S2. Pictures of control efficiency of **MT**/**OMT** (8/2, 0.2 mg/mL) after 1st day (a), 3rd day (b) and 5th day (c) against *P. xylostella* in the greenhouse; Figure S3. Pictures of control efficiency of **MT**/**OMT** (8/2, 0.2 mg/mL)/**CN** after 1st day (a), 3rd day (b) and 5th day (c) against *P. xylostella* in the greenhouse; Figure S4. Pictures of control efficiency of **CK** after 1st day (a), 3rd day (b) and 5th day (c) against *T. urticae* in the greenhouse; Figure S5. Pictures of control efficiency of **MT**/**OMT** (3/7, 0.2 mg/mL) after 1st day (a), 3rd day (b) and 5th day (c) against *T. urticae* in the greenhouse; Figure S6. Pictures of control efficiency of **MT**/**OMT** (3/7, 0.2 mg/mL)/**CN** after 1st day (a), 3rd day (b) and 5th day (c) against *T. urticae* in the greenhouse.

## References

[1] Fu S., Liu Z., Chen J., Sun G., Jiang Y., Li M., Xiong L., Chen S., Zhou Y., Asad M. (2020). Silencing arginine kinase/integrin β1 subunit by transgenic plant expressing dsRNA inhibits the development and survival of *Plutella xylostella*. Pest Manag. Sci..

[2] Vaschetto L.M., Beccacece H.M. (2019). The emerging importance of noncoding RNAs in the insecticide tolerance, with special emphasis on *Plutella xylostella* (Lepidoptera: Plutellidae). Wiley Interdiscip. Rev. RNA.

[3] Banazeer A., Afzal M.B.S., Hassan S., Ijaz M., Shad S.A., Serrão J.E. (2021). Status of insecticide resistance in *Plutella xylostella* (Linnaeus) (Lepidoptera: Plutellidae) from 1997 to 2019: Cross-resistance, genetics, biological costs, underlying mechanisms, and implications for management. Phytoparasitica.

[4] Abdelgaleil S.A., Badawy M.E., Mahmoud N.F., Marei A.E.-S.M. (2019). Acaricidal activity, biochemical effects and molecular docking of some monoterpenes against two-spotted spider mite (*Tetranychus urticae* Koch). Pestic. Biochem. Physiol..

[5] Ndiaye S.G., Welty C. (2022). Augmentation and conservation biological control of *Tetranychus urticae* on hops in Ohio. Biol. Control..

[6] Mavridis K., Papapostolou K.M., Ilias A., Michaelidou K., Stavrakaki M., Roditakis E., Tsagkarakou A., Bass C., Vontas J. (2022). Next-generation molecular diagnostics (TaqMan qPCR and ddPCR) for monitoring insecticide resistance in *Bemisia tabaci*. Pest Manag. Sci..

[7] Shan J., Sun X., Li R., Zhu B., Liang P., Gao X. (2021). Identification of ABCG transporter genes associated with chlorantraniliprole resistance in *Plutella xylostella* (L.). Pest Manag. Sci..

[8] Yamanaka T., Kitabayashi S., Jouraku A., Kanamori H., Kuwazaki S., Sudo M. (2022). A feasibility trial of genomics-based diagnosis detecting insecticide resistance of the diamondback moth. Pest Manag. Sci..

[9] Poonsri W., Pluempanupat W., Chitchirachan P., Bullangpoti V., Koul O. (2015). Insecticidal alkanes from *Bauhinia scandens* var. horsfieldii against Plutella xylostella L. (Lepidoptera: Plutellidae). Ind. Crop. Prod..

[10] Venzon M., Togni P.H.B., Perez A.L., Oliveira J.M. (2020). Control of two-spotted spider mites with neem-based products on a leafy vegetable. Crop Prot..

[11] Fan Q., Li X., Wei C., Wang P., Sun H., Zheng S., Li Y., Tian Z., Liu J., Zhang Y. (2022). Extraction, structure characterization and biological activity determination of (S)-(-)-palasonin from *Butea monosperma* (Lam.) Kuntze seeds. Ind. Crop. Prod..

[12] Li T., Lv M., Wen H., Wang Y., Thapa S., Zhang S., Xu H. (2023). Synthesis of piperine-based ester derivatives with diverse aromatic rings and their agricultural bioactivities against *Tetranychus cinnabarinus* Boisduval, *Aphis citricola* Van der Goot, and *Eriosoma lanigerum* Hausmann. Insects.

[13] Hao M., Xu J., Wen H., Du J., Zhang S., Lv M., Xu H. (2022). Recent advances on biological activities and structural modifications of dehydroabietic acid. Toxins.

[14] Yang R., Lv M., Xu H. (2018). Synthesis of piperine analogs containing isoxazoline/pyrazoline scaffold and their pesticidal bioactivities. J. Agric. Food Chem..

[15] Qu H., Lv M., Yu X., Lian X., Xu H. (2015). Discovery of some piperine-based phenylsulfonylhydrazone derivatives as potent botanically narcotic agents. Sci. Rep..

[16] Koul O., Singh R., Kaur B., Kanda D. (2013). Comparative study on the behavioral response and acute toxicity of some essential oil compounds and their binary mixtures to larvae of *Helicoverpa armigera*, *Spodoptera litura* and *Chilo partellus*. Ind. Crop Prod..

[17] Huang J.-L., Lv M., Xu H. (2017). Semisynthesis of some matrine ether derivatives as insecticidal agents. RSC Adv..

[18] Li S., Sun Z., Zhang B., Lv M., Xu H. (2019). Non-food bioactive products: Semisynthesis, biological activities, and mechanisms of action of oximinoether derivatives of matrine from *Sophora flavescens*. Ind. Crop. Prod..

[19] Xu J., Sun Z., Hao M., Lv M., Xu H. (2020). Evaluation of biological activities, and exploration on mechanism of action of matrine-cholesterol derivatives. Bioorg. Chem..

[20] Zhang B., Xu H. (2019). Research progress of agricultural bioactivities and structural modifications of matrine and its analogues. Chin. J. Pestic. Sci..

[21] Lv M., Ma Q., Zhang S., Xu H. (2021). Agrochemical properties evaluation of some imines alkaloids of matrine/oxymatrine. Bioorganic Med. Chem. Lett..

[22] Xu H., Xu M., Sun Z., Li S. (2019). Preparation of matrinic/oxymatrinic amide derivatives as insecticidal/acaricidal agents and study on the mechanisms of action against *Tetranychus cinnabarinus*. J. Agric. Food Chem..

[23] Ding Y., Li N., Sun J., Zhang L., Guo J., Hao X., Sun Y. (2019). Oxymatrine inhibits bocavirus MVC replication, reduces viral gene expression and decreases apoptosis induced by viral infection. Virol. Sin..

[24] Lan X., Zhao J., Zhang Y., Chen Y., Liu Y., Xu F. (2020). Oxymatrine exerts organ- and tissue-protective effects by regulating in-flammation, oxidative stress, apoptosis, and fibrosis: From bench to bedside. Pharmacol. Res..

[25] Chen K., Zhu P., Ye J., Liao Y., Du Z., Chen F., Juanjuan H., Zhang S., Zhai W. (2019). Oxymatrine inhibits the migration and invasion of hepatocellular carcinoma cells by reducing the activity of MMP-2/-9 via regulating p38 signaling pathway. J. Cancer.

[26] Arena J.S., Merlo C., Defagó M.T., Zygadlo J.A. (2019). Insecticidal and antibacterial effects of some essential oils against the poultry pest *Alphitobius diaperinus* and its associated microorganisms. J. Pest Sci..

[27] Sun Y., Cai X., Cao J., Wu Z., Pan D. (2018). Effects of 1,8-cineole on carbohydrate metabolism related cell structure changes of salmonella. Front. Microbiol..

[28] Meng C., Zeng W., Lv J., Wang Y., Gao M., Chang R., Li Q., Wang X. (2021). 1,8-cineole ameliorates ischaemic brain damage via TRPC6/CREB pathways in rats. J. Pharm. Pharmacol..

[29] de Albuquerque Lima T., de Queiroz Baptista N.M., de Oliveira A.P., da Silva P.A., de Gusmão N.B., dos Santos Correia M.T., Napoleão T.H., da Silva M.V., Paiva P.M. (2021). Insecticidal activity of a chemotype VI essential oil from *Lippia alba* leaves collected at Caatinga and the major compound (1,8-cineole) against *Nasutitermes corniger* and *Sitophilus zeamais*. Pestic. Biochem. Physiol..

[30] Kumrungsee N., Pluempanupat W., Koul O., Bullangpoti V. (2014). Toxicity of essential oil compounds against diamondback moth, *Plutella xylostella*, and their impact on detoxification enzyme activities. J. Pest Sci..

[31] Liao M., Li S., Wu H., Gao Q., Shi S., Huang Y., Cao H. (2022). Transcriptomic analysis of *Sitophilus zeamais* in response to limonene fumigation. Pest. Manag. Sci..

[32] Born F.D., da Camara C.A., de Melo J.P., de Moraes M.M. (2018). Acaricidal property of the essential oil from *Lippia gracilis* against *Tetranychus urticae* and a natural enemy, *Neoseiulus californicus*, under greenhouse conditions. Exp. Appl. Acarol.

[33] Kavallieratos N.G., Nika E.P., Skourti A., Boukouvala M.C., Ntalaka C.T., Maggi F., Spinozzi E., Petrelli R., Perinelli D.R., Benelli G. (2022). *Carlina acaulis* essential oil nanoemulsion as a new grain protectant against different developmental stages of three stored-product beetles. Pest Manag. Sci..

[34] Ebadollahi A. (2016). Chemical composition, acaricidal and insecticidal effects of essential oil from *Achillea filipendulina* against two arthropod pests; *Oryzaephilus surinamensis* and *Tetranychus urticae*. Toxin Rev..

[35] Ebadollahi A., Sendi J.J., Aliakbar A. (2017). Efficacy of Nanoencapsulated *Thymus eriocalyx* and *Thymus kotschyanus* Essential Oils by a Mesoporous Material MCM-41 Against *Tetranychus urticae* (Acari: Tetranychidae). J. Econ. Èntomol..

[36] Ebadollahi A., Sendi J.J. (2017). Acaricidal potentials of the terpene-rich essential oils of two Iranian *Eucalyptus* species against *Tetranychus urticae* Koch. J. Oleo Sci..

[37] Huang X., Huang Y., Yang C., Liu T., Liu X., Yuan H. (2021). Isolation and insecticidal activity of essential oil from *Artemisia la-vandulaefolia* DC. against *Plutella xylostella*. Toxins.

[38] Tak J.H., Isman M.B. (2015). Enhanced cuticular penetration as the mechanism for synergy of insecticidal constituents of rosemary essential oil in *Trichoplusia ni*. Sci. Rep..

[39] Aungtikun J., Soonwera M., Sittichok S. (2021). Insecticidal synergy of essential oils from *Cymbopogon citratus* (Stapf.), *Myristica fragrans* (Houtt.), and *Illicium verum* Hook. f. and their major active constituents. Ind. Crop. Prod..

[40] Sun Z., Lv M., Huang W., Li T., Xu H. (2022). Development of botanical pesticides: Exploration on the phenotype of vestigial wings of insect pests induced by plant natural products or their derivatives by blocking tyrosine phosphorylation of insulin receptor 1. J. Agric. Food Chem..

[41] Cui G., Yuan H., He W., Deng Y., Sun R., Zhong G. (2021). Synergistic effects of botanical curcumin-induced programmed cell death on the management of *Spodoptera litura* Fabricius with avermectin. Ecotoxicol. Environ. Saf..

[42] Gao Q., Shi Y., Liao M., Xiao J., Li X., Zhou L., Liu C., Liu P., Cao H. (2019). Laboratory and field evaluation of the aphidicidal activity of moso bamboo (*Phyllostachys pubescens*) leaf extract and identification of the active components. Pest Manag. Sci..

[43] Lu X., Liu J., Weng H., Ma Z., Zhang X. (2019). Efficacy of binary combinations between methyl salicylate and carvacrol against thrips *Anaphothrips obscurus*: Laboratory and field trials. Pest Manag. Sci..

[44] Nong Q.-Y., Liu Y.-A., Qin L.-T., Liu M., Mo L.-Y., Liang Y.-P., Zeng H.-H. (2020). Toxic mechanism of three azole fungicides and their mixture to green alga *Chlorella pyrenoidosa*. Chemosphere.

[45] Tak J.-H., Jovel E., Isman M.B. (2015). Comparative and synergistic activity of *Rosmarinus officinalis* L. essential oil constituents against the larvae and an ovarian cell line of the cabbage looper, *Trichoplusia ni* (Lepidoptera: Noctuidae). Pest Manag. Sci..

[46] Tak J.H., Isman M.B. (2017). Acaricidal and repellent activity of plant essential oil-derived terpenes and the effect of binary mixtures against *Tetranychus urticae* Koch (Acari: Tetranychidae). Ind. Crop Prod..

[47] Benelli G., Pavela R., Iannarelli R., Petrelli R., Cappellacci L., Cianfaglione K., Afshar F.H., Nicoletti M., Canale A., Maggi F. (2017). Synergized mixtures of Apiaceae essential oils and related plant-borne compounds: Larvicidal effectiveness on the filariasis vector *Culex quinquefasciatus* Say. Ind. Crop. Prod..

[48] Yuan L., Yang X., Yu X., Wu Y., Jiang D. (2018). Resistance to insecticides and synergistic and antagonistic effects of essential oils on dimefluthrin toxicity in a field population of *Culex quinquefasciatus* Say. Ecotoxicol. Environ. Saf..

[49] Zhou C., Yang H., Wang Z., Long G.-Y., Jin D.-C. (2019). Protective and detoxifying enzyme activity and ABCG subfamily gene expression in *Sogatella furcifera* under insecticide stress. Front. Physiol..

[50] Li Y., Sun H., Tian Z., Li Y., Ye X., Li R., Li X., Zheng S., Liu J., Zhang Y. (2021). Identification of key residues of carboxylesterase PxEst-6 involved in pyrethroid metabolism in *Plutella xylostella* (L.). J. Hazard. Mater..

[51] Zhu W., Duan Y., Chen J., Merzendorfer H., Zou X., Yang Q. (2022). SERCA interacts with chitin synthase and participates in cu-ticular chitin biogenesis in *Drosophila*. Insect. Biochem. Mol. Biol..

[52] Xu H., Zhang K., Lv M., Hao M. (2021). Construction of cholesterol oxime ether derivatives containing isoxazoline/isoxazole fragments and their agricultural bioactive properties/control efficiency. J. Agric. Food Chem..

[53] Wu C., Yu X., Wang B., Liu J., Meng F., Zhao Y., Xiong L., Yang N., Li Y., Li Z. (2020). Synthesis, insecticidal evaluation, and 3D-QASR of novel anthranilic diamide derivatives containing N-arylpyrrole as potential ryanodine receptor activators. J. Agric. Food Chem..

[54] Sun Y.P., Johnson E.R. (1960). Analysis of joint action of insecticides against house flies. J. Econ. Entomol..

[55] Nian X., He Y., Lu L., Zhao R. (2015). Evaluation of alternative *Plutella xylostella* control by two *Isaria fumosorosea* conidial formu-lations-oil-based formulation and wettable powder-combined with *Bacillus thuringiensis*. Pest Manag. Sci..

[56] Hao M., Sun Z., Xu J., Lv M., Xu H. (2020). Semisynthesis and pesticidal activities of derivatives of the diterpenoid andrographolide and investigation on the stress response of *Aphis citricola* Van der Goot (Homoptera: Aphididae). J. Agric. Food Chem..

[57] Bradford M.M. (1976). A rapid and sensitive method for the quantitation of microgram quantities of protein utilizing the principle of protein-dye binding. Anal. Biochem..

[58] Li T., Lv M., Wen H., Wang J., Wang Z., Xu J., Fang S., Xu H. (2022). High value-added application of natural plant products in crop protection: Construction and pesticidal activities of piperine-type ester derivatives and their toxicology study. J. Agric. Food Chem..

[59] Zhou H., Liu S., Wan F., Jian Y., Guo F., Chen J., Ning Y., Ding W. (2021). Graphene oxide–acaricide nanocomposites advance acaricidal activity of acaricides against *Tetranychus cinnabarinus* by directly inhibiting the transcription of a cuticle protein gene. Environ. Sci. Nano.

[60] Togawa T., Dunn A., Emmons A.C., Willis J.H. (2007). CPF and CPFL, two related gene families encoding cuticular proteins of *Anopheles gambiae* and other insects. Insect Biochem. Mol. Biol..

