# Supplementary material for: Exploration of Synergistic Pesticidal Activities, Control Effects and Toxicology Study of a Monoterpene Essential Oil with Two Natural Alkaloids
Source: Toxins (Basel). 2023 Mar 25;15(4):240. doi: 10.3390/toxins15040240 (PMC10142011; doi:10.3390/toxins15040240)
Supplement: Supplementary file 1 [file toxins-15-00240-s001.zip › toxins-2134122-supplementary.pdf]

toxins

MDPI

Supplementary Information

Exploration of Synergistic Pesticidal Activities, Control Effects and Toxicology Study of a Monoterpene Essential Oil with Two Natural Alkaloids

Jianwei Xu, Min Lv, Shanshan Fang, Yanyan Wang, Houpeng Wen, Shaoyong Zhang and Hui Xu

1. Biological assay

1.1. Insecticidal activity of matrine (MT), oxymatrine (OMT) and their binary complexes against P. xylostella by the leaf-loading poison method

The solutions of matrine (**MT**), oxymatrine (**OMT**) and their binary complexes were prepared in acetone at 10 mg/mL (toosendanin as the positive control and acetone as CK) (Table 1). For each compound, 45 robust third instar larvae of *P. xylostella* were selected out (15 insects per group). The corresponding solution (1 μL) was evenly spread on a cabbage leaf disc (surface area: 0.25 cm^2). One piece of the above discs was added and eaten up by each *P. xylostella*, which was raised in each well of 12-well culture plates during 48 h (temperature: 25 ± 2 °C; RH: 70 ± 10%; photoperiod: light/dark = 16/8 h). Their corrected mortality rate (CMR) values (%) = $(T - C) \times 100/(100\% - C)$; C is the mortality rate of CK, and T is the mortality rate of the treated *P. xylostella*.

1.2. Insecticidal activity of MT, OMT, MT/OMT (8/2), 1,8-cineole (CN) and MT/OMT (8/2)/CN against P. xylostella by the leaf-dipping method

The procedure for evaluation of larvicidal activity of 1,8-cineole (**CN**), **MT/OMT** (8/2), and **MT/OMT** (8/2)/**CN** against *P. xylostella* (Fig. 2) was as follows: The solutions of 1,8-cineole (**CN**, 0.4 and 0.5 mg/mL), **MT/OMT** (8/2, 0.5 mg/mL) , and **MT/OMT** (8/2, 0.5 mg/mL)/**CN** (C_{CN} = 0.4 mg/mL) were prepared in acetone (acetone was used as CK). For each compound, 45 robust third instar larvae of *P. xylostella* were selected out (15 insects per group). The cabbage leaf disc (surface area: 0.25 cm^2).was dipped into the corresponding solution for 3 s and taken out. The treated ones were added to three dishes during 48 h (15 insects per dish) (temperature: 25 ± 2 °C; RH: 70 ± 10%; photoperiod: light/dark = 16/8 h). Their CMR values were calculated in the same way as mentioned above.

The procedure for determination of LC_{50} values of **MT**, **OMT**, **MT**/**OMT** (8/2), and **MT**/**OMT** (8/2)/**CN** at 48 h against *P. xylostella* was as follows (Table 2): Firstly, five different concentrations (2, 1, 0.5, 0.25, and 0.125 mg/mL) of **MT**, **OMT**, **MT**/**OMT** (8/2), and **MT**/**OMT** (8/2)/**CN** were prepared in acetone (acetone was used as CK). Five concentrations of β-cypermethrin (a positive control) were set as 0.5, 0.25, 0.125, 0.0625, and 0.03125 mg/mL in acetone, respectively. For each concentration, 45 robust third instar larvae of *P. xylostella* were selected out (15 insects per group). The cabbage leaf disc (surface area: 0.25 cm^2) was dipped into the corresponding solution for 3 s and taken out. The treated ones were added to three dishes during 48 h (15 insects per dish) (temperature: 25 ± 2 °C; RH: 70 ± 10%; photoperiod: light/dark = 16/8 h). Their CMR values at 48 h were calculated in the same way as mentioned above. Finally, their 48 h median lethal concentration (LC_{50}) values were calculated upon the concentrations and CMRs (Table 3).

The co-toxicity coefficient (CTC) values of the binary complexes were further evaluated according to Sun's formula. The value of CTC is used to determine whether the efficiency is increased: when CTC > 120, it is synergistic; when CTC < 80, it is antagonistic; when 80 < CTC < 120, it is additive. A significant synergistic effect is observed when the value of CTC is 200.

1.3. Control efficiency of MT/OMT (8/2), and MT/OMT (8/2)/CN against P. xylostella in the greenhouse

The solutions of **MT**/**OMT** (8/2), **MT**/**OMT** (8/2)/**CN** and β-cypermethrin were prepared at 0.2 mg/mL in 0.1% aq. Tween-80, respectively (Table 4). Each cabbage seedling was infested with 20 third instar larvae of *P. xylostella* prior to spraying. One cabbage seedling was chosen for one group, and each treatment was three replicates. An airbrush was used to spray 10 mL of the corresponding solution for each treatment. The cabbage seedlings treated with 0.1% aq. Tween-80 alone were used as CK (temperature: 25 ± 2 °C; RH: 70 ± 10%; photoperiod: light/dark = 16/8 h). Their control effects on the 1st, 3rd, and 5th days were calculated in the same way as mentioned above.

1.4. Acaricidal activity of MT, OMT, CN, and their mixtures against Tetranychus urticae by the slide-dipping method

The solutions of **MT**, **OMT**, their binary complexes (with different mass ratio), and spirodiclofen (a positive control) (treated by 0.1 g/L of aq. Tween-80 as CK) were prepared at 0.5 mg/mL in Tween-80 in water (0.1 g/L), respectively (Table 5). For each compound, 90–120 healthy and size-consistency female adults of mites (30–40 ones per group) were selected out. Slides affixed with mites were dipped into the corresponding solution for 5 s and taken out (temperature: 26 ± 1 °C; RH: 60–80%; photoperiod: light/dark = 14/10 h). Their mortalities at 48 and 72 h were calculated as follows: corrected mortality rate (%) = (T − C) × 100/(100% − C); C is the mortality rate of CK, and T is the mortality rate of the treated *T. urticae*.

According to the above results, the acaricidal activity of **CN**, and **MT/OMT** (3/7)/**CN** against *P. xylostella* (Table 5) was tested as follows: The solution of **CN** was prepared at 0.048 mg/mL in Tween-80 in water (0.1 g/L), and the solution of **MT/OMT** (3/7)/**CN** was prepared at 0.5 mg/mL in Tween-80 in water (0.1 g/L) containing **CN** (C_{CN} = 0.048 mg/mL). The next procedure was done in the same way as mentioned above.

1.5. LC_{50} of MT, OMT, MT/OMT (3/7), and MT/OMT (3/7)/CN against T. urticae

Firstly, five different concentrations (6, 3, 1.5, 0.75, and 0.375 mg/mL) of **MT** and **OMT** were prepared in Tween-80 in water (0.1 g/L) (treated by 0.1 g/L of aq. Tween-80 as CK) (Table 6). Six concentrations of **MT/OMT** (3/7), and **MT/OMT** (3/7)/**CN** were set as 4, 2, 1, 0.5, 0.25, and 0.125 mg/mL in Tween-80 in water (0.1 g/L), respectively. Five concentrations of spirodiclofen were set as 0.5, 0.25, 0.125, 0.0625, and 0.03125 mg/mL in Tween-80 in water (0.1 g/L), respectively. For each concentration, 90–120 healthy and size-consistency female adults of mites (30–40 ones per group) were selected out. Slides affixed with mites were dipped into the corresponding solution for 5 s and taken out (temperature: 26 ± 1 °C; RH: 60–80%; photoperiod: light/dark = 14/10 h). Their CMR values at 72 h were calculated in the same way as mentioned above. Finally, LC_{50} values were calculated by the linear regressions of 72 h CMRs (%)and concentrations (Table 7). The co-toxicity coefficient (CTC) values of the binary complexes were further evaluated according to Sun's formula.

1.6. Control efficiency of MT/OMT (3/7), and MT/OMT (3/7)/CN against T. urticae in the greenhouse

The solutions of **MT/OMT** (3/7), **MT/OMT** (3/7)/**CN**, and spirodiclofen were all prepared at 0.2 mg/mL in 0.1% aq. Tween-80, respectively. Asparagus bean plants were infested with the female adults of *T. urticae* prior to spraying. Three plants were chosen for one group, and each treatment was three replicates. An airbrush was used to spray 10 mL of the corresponding solution for three replicates. The plants treated with 0.1% aq. Tween-80 alone were used as CK (temperature: 26 ± 1 °C; RH: 60–80%; photoperiod: light/dark = 14/10 h). Their control effects on the 1st, 3rd, and 5th days were calculated in the same way as mentioned above (Table 8).

1.7. Enzyme activity assay

1.7.1. Sample preparation

According to the above-mentioned leaf-dipping method. 180 robust 3rd instar larvae of *P. xylostella* were treated with **CN**, **MT/OMT** (8/2) and **MT/OMT** (8/2)/**CN** at 0.4 mg/mL, respectively (treated by acetone as CK). 30 surviving larvae in the treated group were collected at 12, 24, 36, and 48 h, respectively. They were then snap-frozen in liquid nitrogen and stored at -80 °C for subsequent enzyme activity analysis.

1.7.2. Preparation of homogenous liquid

The homogenization treatment (including ten larvae: Weight (g)/Volume (mL) = 1/10) was performed at ice bath. The homogenous liquid was obtained for carboxylesterase (CarE) activity assay when the sample was centrifuged at 12000 g for 30 min at 4 °C. The homogenous liquid was obtained for glutathione-S-transferase (GST) activity assay when the sample was centrifuged at 8000 g for 10 min at 4 °C.

1.7.3. CarE and GST activity assay

The absorption values over time of CarE and GST were tested by using of CarE (α-naphthyl acetate (α-NA) as a substrate) and GST (1-chloro-2,4-dinitrobenzene and reduced glutathione as substrates) assay kits (Suzhou Keming Biotechnology Co., Ltd., China), respectively. Total protein concentration was determined according to the Bradford method (using bovine serum albumin (BSA) as a standard). The protein content was tested by BCA protein quantitative assay kit (Shaanxi Zhonghui Hecai Biomedical Technology Co., Ltd., China). Finally, the enzymes activity values were obtained according to the absorption value and the protein content. Each treatment was replicated three times.

2. Pictures of control effects of different complexes against *P. xylostella*

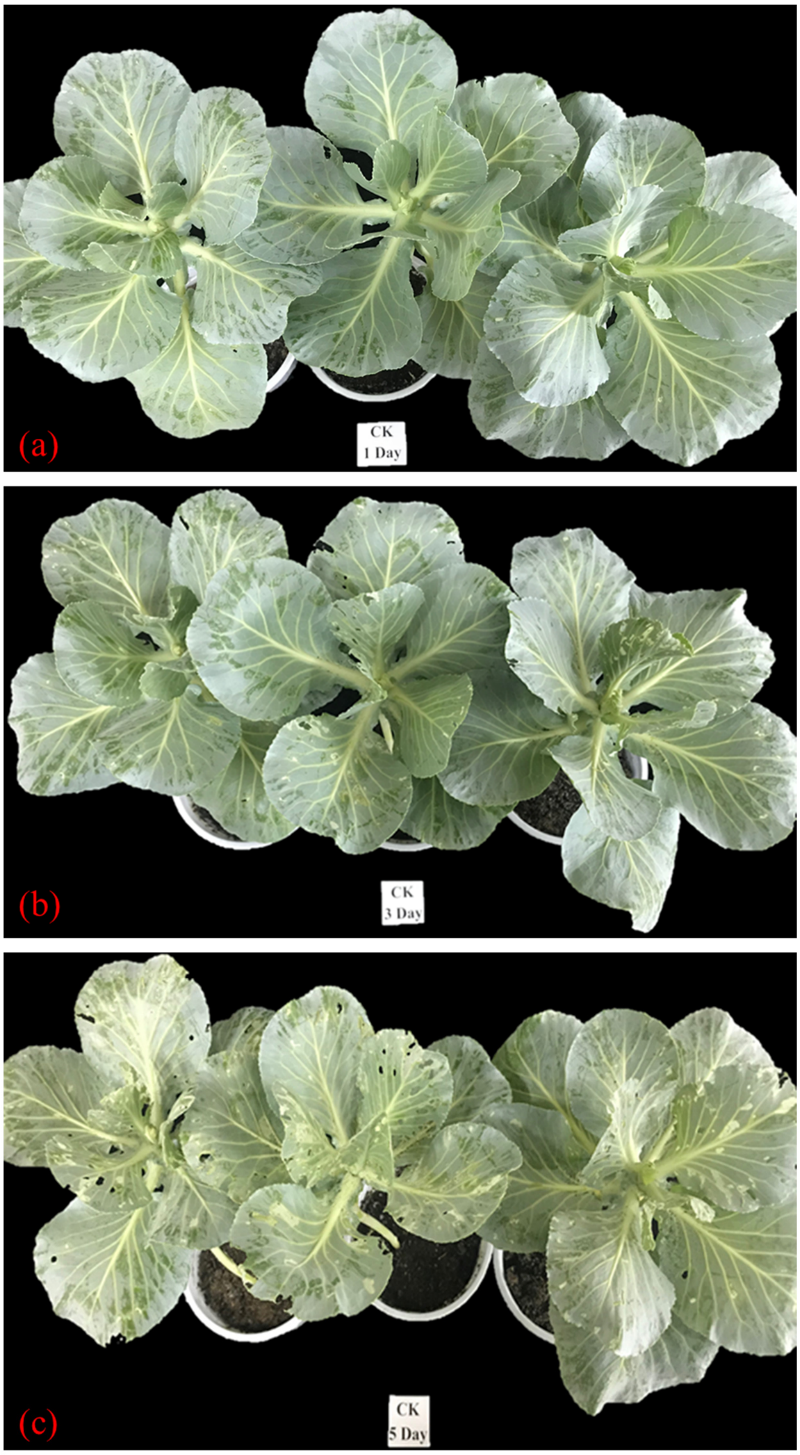

Figure S1. Pictures of control efficiency of **CK** after 1st day (**a**), 3rd day (**b**) and 5th day (**c**) against *P. xylostella* in the greenhouse.

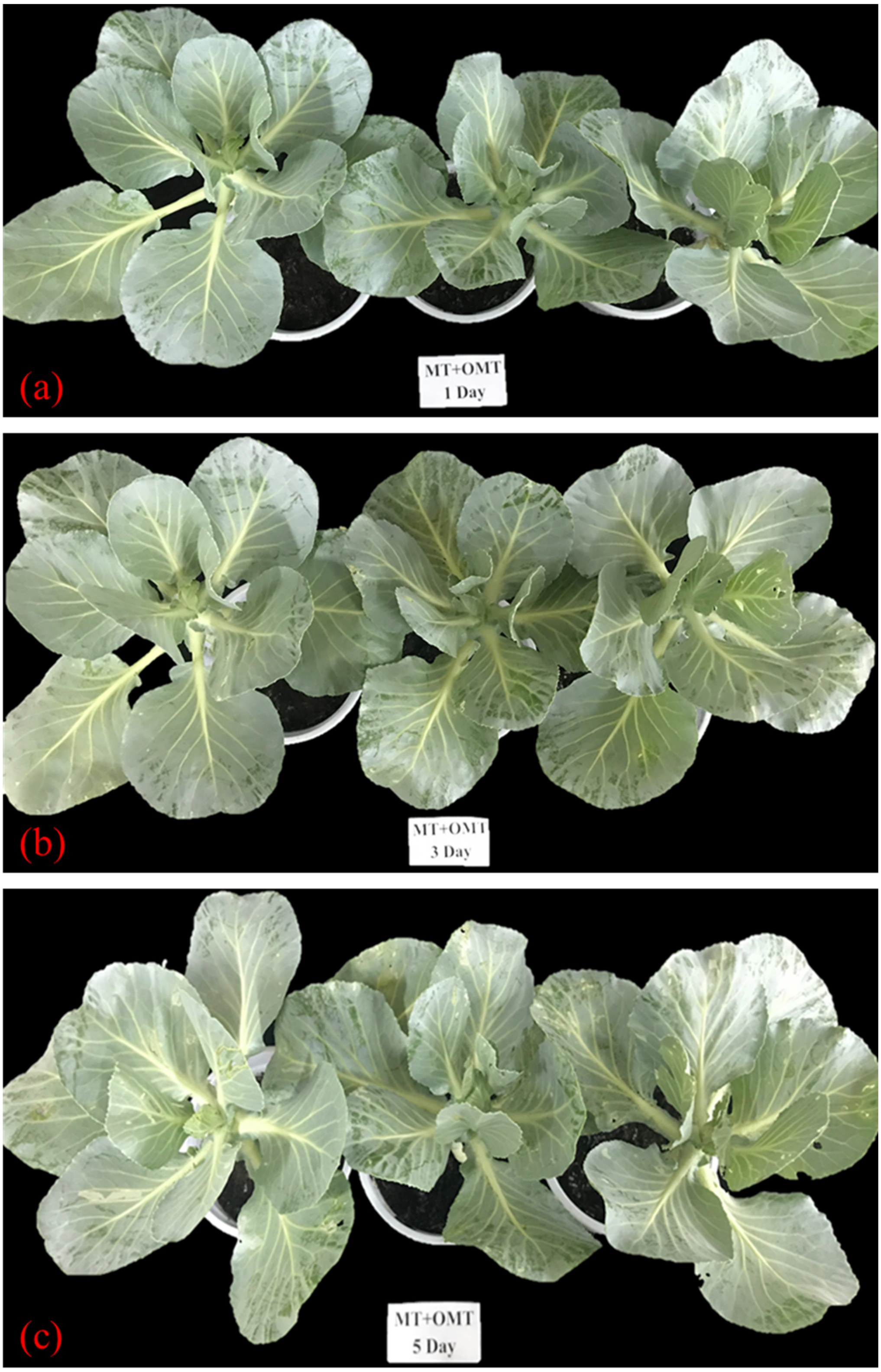

Figure S2. Pictures of control efficiency of **MT/OMT** (8/2, 0.2 mg/mL) after 1st day (**a**), 3rd day (**b**) and 5th day (**c**) against *P. xylostella* in the greenhouse.

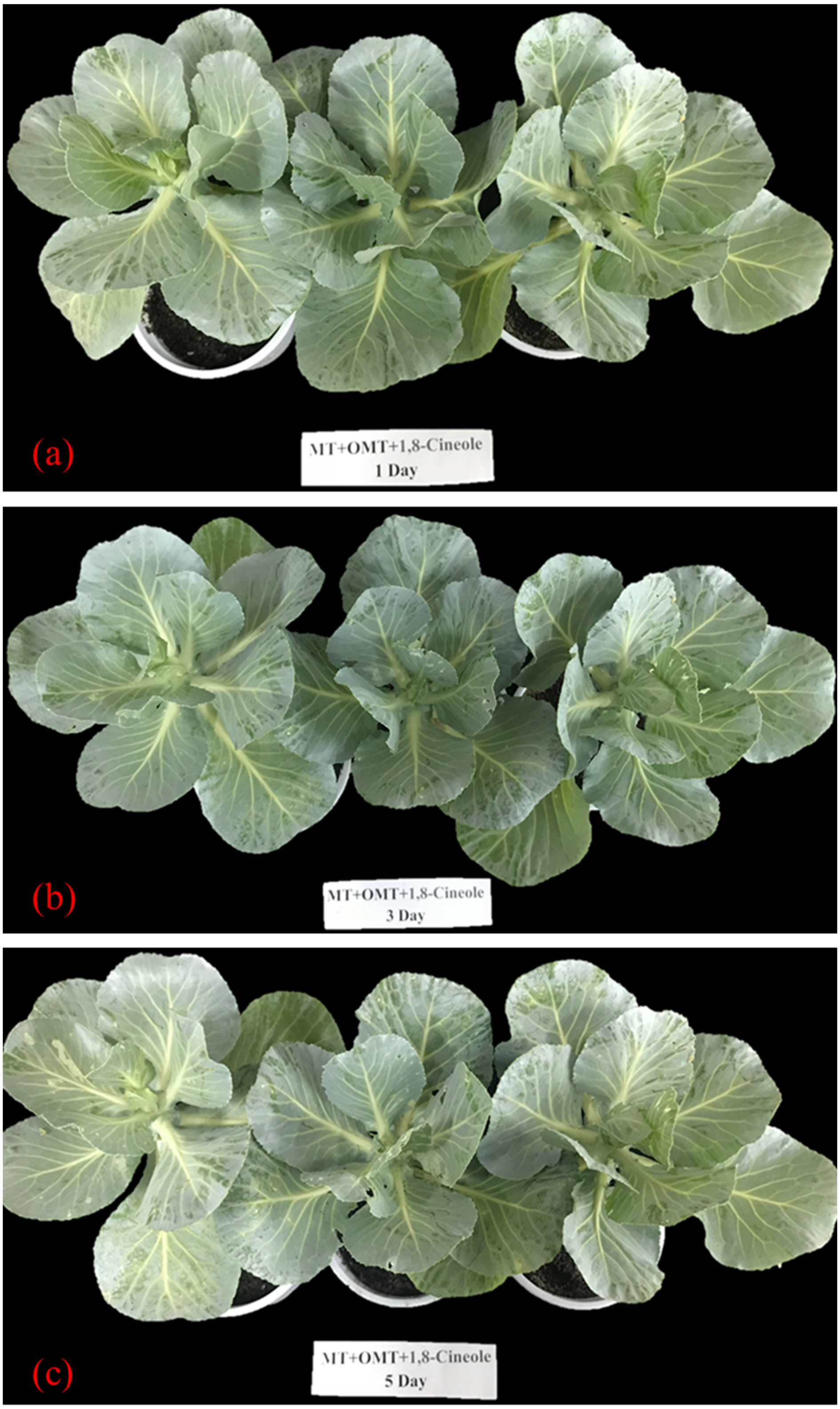

Figure S3. Pictures of control efficiency of **MT/OMT** (8/2, 0.2 mg/mL)/**CN** after 1st day (a), 3rd day (b) and 5th day (c) against *P. xylostella* in the greenhouse.

3. Pictures of control effects of different complexes against *T. cinnabarinus*

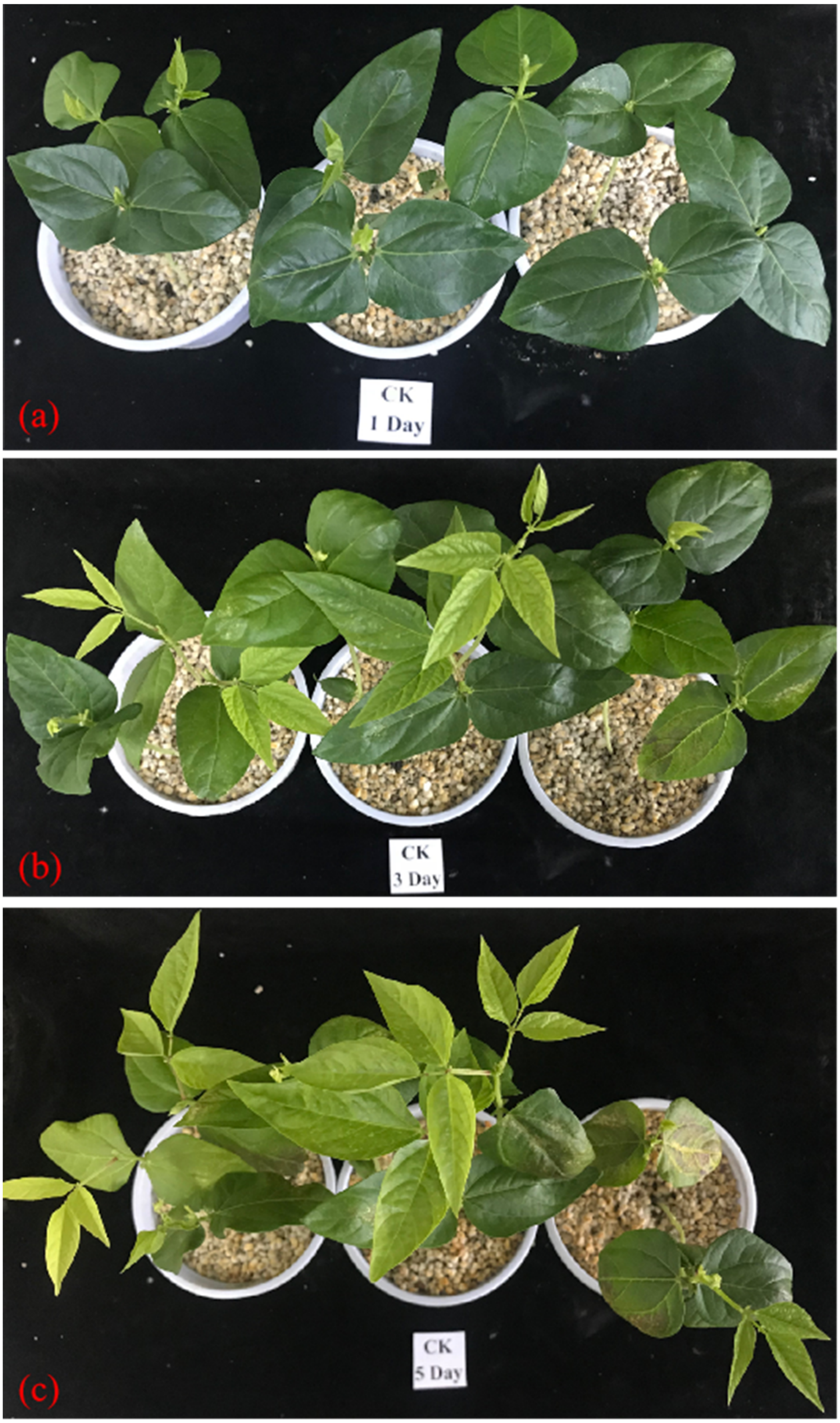

Figure S4. Pictures of control efficiency of **CK** after 1st day (a), 3rd day (b) and 5th day (c) against *T. urticae* in the greenhouse.

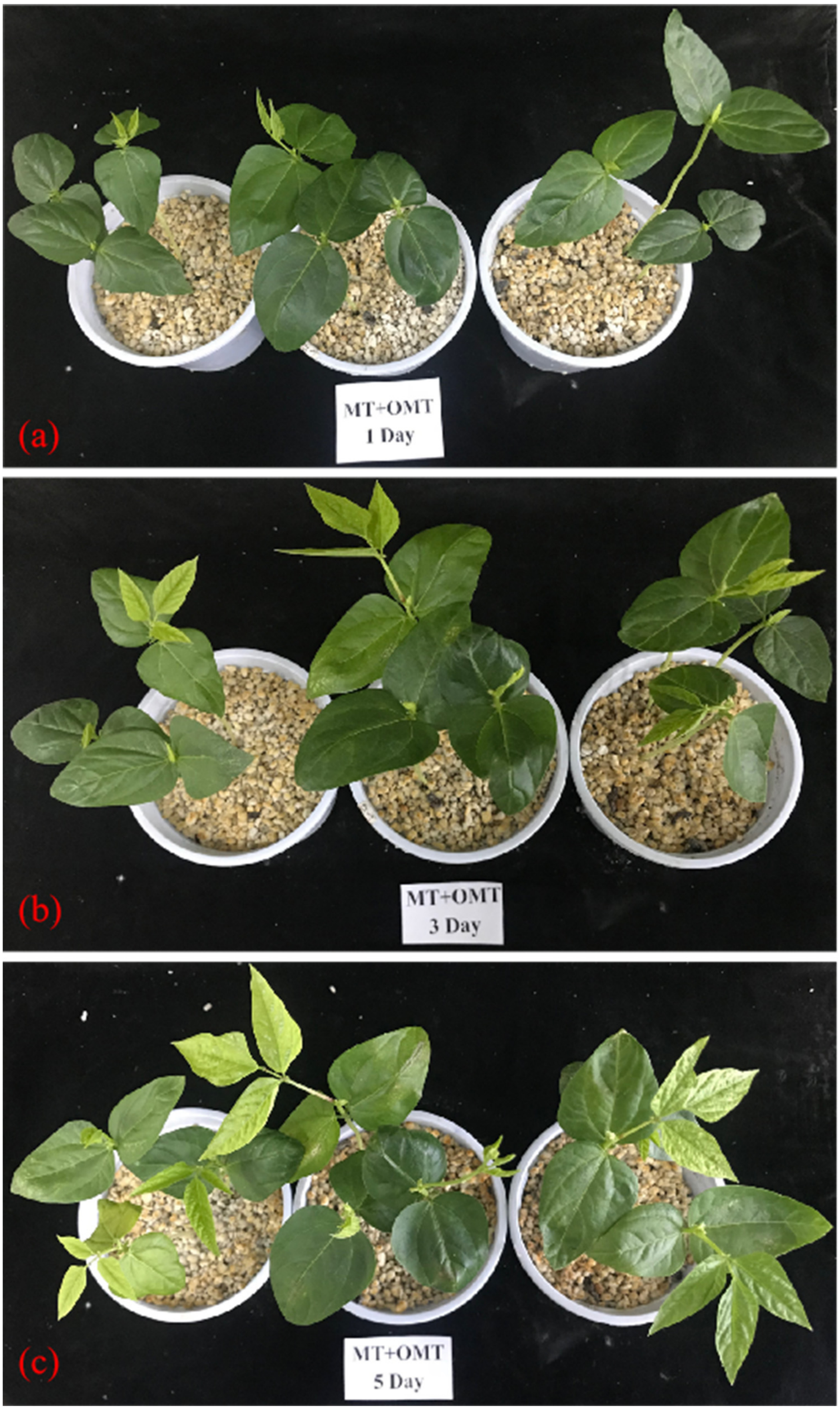

Figure S5. Pictures of control efficiency of **MT/OMT** (3/7, 0.2 mg/mL) after 1st day (a), 3rd day (b) and 5th day (c) against *T. urticae* in the greenhouse.

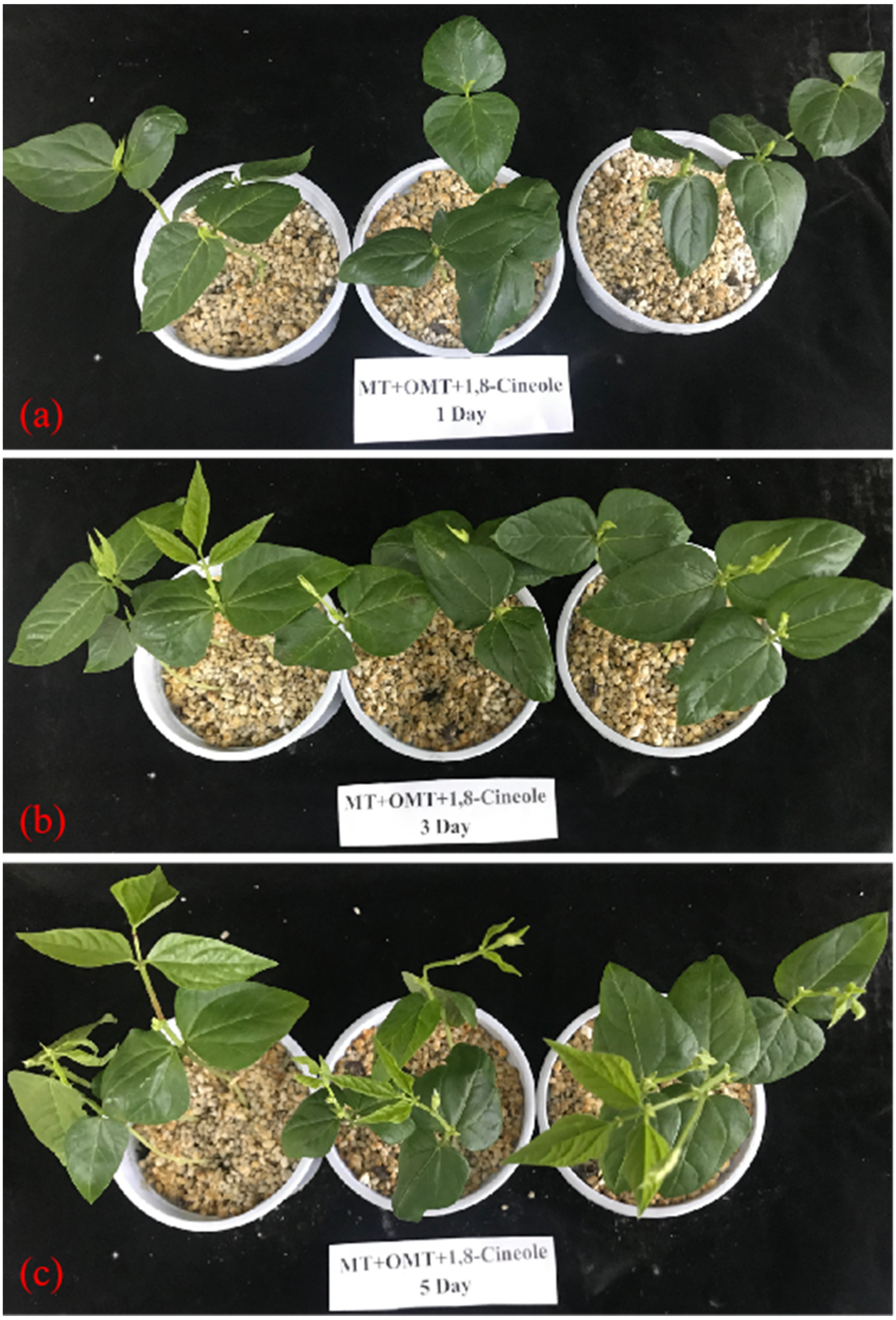

Figure S6. Pictures of control efficiency of **MT/OMT** (3/7, 0.2 mg/mL)/**CN** after 1st day (a), 3rd day (b) and 5th day (c) against *T. urticae* in the greenhouse.
